# Exploring the molecular crosstalk between the sex steroids drospirenone and ethinylestradiol with vaginal lactobacilli

**DOI:** 10.3389/fmicb.2026.1725887

**Published:** 2026-04-09

**Authors:** Aryanna Muscò, Chiara Tarracchini, Sonia Mirjam Rizzo, Alice Viappiani, Marco Ventura, Francesca Turroni

**Affiliations:** 1 Laboratory of Probiogenomics, Department of Chemistry, Life Sciences, and Environmental Sustainability, University of Parma, Parma, Italy; 2 GenProbio s.r.l., Parma, Italy; 3 Interdepartmental Research Center “Microbiome Research Hub”, University of Parma, Parma, Italy

**Keywords:** *Lactobacillus*, *Lactobacillus crispatus*, sex hormones, transcriptomic (RNA-seq), vaginal microbiota

## Abstract

**Introduction:**

The vaginal microbiota, dominated by the genus *Lactobacillus* spp., plays a crucial role in maintaining vaginal homeostasis and protecting against infection through lactic acid production, antimicrobial compound secretion and competitive exclusion of pathogens. Although hormonal fluctuations are known to influence microbial composition, the molecular mechanisms underlying these interactions remain largely unexplored. The aim of this study was to investigate the direct effects of the synthetic sex hormones drospirenone and ethinylestradiol, key components of hormonal contraceptives, on representative vaginal *Lactobacillus* species.

**Methods:**

Representative *Lactobacillus* species associated with different vaginal Community State Types (CSTs) were exposed to drospirenone and ethinylestradiol under simulated vaginal conditions. Lactobacilli responses were assessed using growth assays and RNA-seq transcriptome profiling to evaluate species-specific transcriptional changes following hormonal exposure.

**Results:**

Among the tested strains, *Lactobacillus crispatus* PRL2021 showed the most pronounced transcriptomic modulation. In this strain, hormone treatment led to the upregulation of genes involved in cell wall biosynthesis, amino acid and carbohydrate metabolism, and stress adaptation. Specifically, expression of the histidine kinase gene *sasA_1*, part of a two-component regulatory system potentially involved in environmental sensing, was induced. Additionally, the *ribBA* and *ribE* genes, predicted to be involved in riboflavin biosynthesis and associated with antioxidant defense and mucosal protection, were upregulated.

**Conclusion:**

These findings demonstrate that drospirenone and ethinylestradiol can directly modulate bacterial gene expression, revealing a previously underinvestigated molecular crosstalk between host endocrine signals and the vaginal microbiota. This interaction may contribute to the maintenance of vaginal eubiosis and has potential implications for the development of microbiome-targeted strategies to support women’s health. Further studies are needed to elucidate the broader functional consequences of hormone–microbiota interactions and their clinical relevance.

## 1 Introduction

The vaginal microbiota, in contrast to other microbial communities colonizing the human body, is characterized by low microbial diversity, with a predominance of *Lactobacillus* species (Gajer et al., 2012; Ravel et al., 2011). Metagenomic analyses of large cohorts of healthy individuals have shown that the most prevalent vaginal microbial communities can be categorized into five Community State Types (CSTs), four of which are dominated by a single *Lactobacillus* species: *Lactobacillus crispatus* (CST I), *Lactobacillus gasseri* (CST II), *Lactobacillus iners* (CST III), and *Lactobacillus jensenii* (CST V) (Mancabelli et al., 2020, 2021). Recent evidence shows that CST I is more strongly associated with vaginal health than other CSTs, suggesting that *L. crispatus* could potentially serve as a microbial biomarker for a healthy vaginal microbiota (Lepargneur, 2016; DiGiulio et al., 2015; Petrova et al., 2015). This association may be attributed to the ability of *L. crispatus* to produce various antimicrobial compounds (Borges et al., 2014; Kaewsrichan et al., 2006), to maintain the integrity of vaginal immune barriers, and to reduce levels of proinflammatory cytokines, which are typically elevated following bacterial vaginosis (BV) (Rose et al., 2012; Doerflinger et al., 2014). The composition of the vaginal microbiota is influenced by a variety of physiological and environmental factors, including menstruation (Kaur et al., 2020; Gajer et al., 2012; Eschenbach et al., 2000; Lambert et al., 2013; Santiago et al., 2012), use of hormonal contraceptives (Bastianelli et al., 2021; Madden et al., 2012), pregnancy (Li et al., 2025), menopause (Brotman et al., 2018), and sexual activity (Eschenbach et al., 2001). These observations underscore the critical role of hormonal fluctuations in modulating the vaginal microbial ecosystem (Song et al., 2020). Elevated estrogen levels have been positively associated with the maintenance of a *Lactobacillus*-dominated microbiota (Bastianelli et al., 2021). Specifically, circulating estradiol levels stimulate glycogen accumulation in the vaginal epithelium, which has been shown to promote the growth of lactobacilli, including *L. crispatus* (Acharya et al., 2021), facilitating bacterial adhesion to vaginal epithelial cells (Clabaut et al., 2021). Multiple studies have demonstrated that administration of female sex hormones, whether oral or systemic, supports vaginal eubiosis (i.e., healthy and balanced vaginal microbiota) characterized by *Lactobacillus*-dominated communities (Bastianelli et al., 2021; Madden et al., 2012), thanks to their lactic acid production and consequently pH reduction, antimicrobial compound secretion, and competitive exclusion of pathogens. This effect is observed both when estrogen is administered alone and when co-administered with progesterone, resulting in enhanced microbial stability across reproductive-aged, perimenopausal, and postmenopausal women. In contrast, progestin-only regimens have shown variable outcomes and appear less effective at supporting *Lactobacillus*-dominant vaginal microbiota (Ratten et al., 2021).

Ethinylestradiol (EE) is a synthetic estrogen widely used in hormonal contraceptives due to its high oral bioavailability and metabolic stability, which allow effective suppression of ovulation through modulation of the hypothalamic–pituitary–ovarian axis (Machado et al., 2011). Drospirenone (DRSP) is a fourth-generation synthetic progestin structurally related to spironolactone and characterized by anti-androgenic and anti-mineralocorticoid properties, distinguishing it from earlier progestins (Oelkers, 2004). The combination of EE and DRSP is commonly prescribed in systemic contraceptive regimens and exerts systemic endocrine effects (Machado et al., 2011). Considering that such combined contraceptive may indirectly, or potentially directly, influence mucosal environments, including the vaginal ecosystem, *in vitro* studies have shown that sex steroids, including estradiol and progesterone, can influence bacterial growth, biofilm formation, and metabolite production in gut-associated bacteria, suggesting analogous mechanisms may apply to vaginal *Lactobacillus* species (Hammouda et al., 2023). It is well established that estrogens promote glycogen accumulation in vaginal epithelial cells, which, upon degradation by α-amylase, generate substrates that can be metabolized by lactobacilli into lactic acid, thereby contributing to a eubiotic vaginal environment (Amabebe and Anumba, 2018; Petrova et al., 2015; Borrego-Ruiz and Borrego, 2025). Although the molecular mechanisms by which hormones directly modulate the vaginal microbiota remain incompletely understood, hormone–microorganism interactions are already documented in the literature. Some studies examine how the transcriptome of microorganisms, particularly bifidobacteria, is modulated by interactions with hormones, specifically melatonin and insulin present in breast milk (Rizzo et al., 2023, 2025).

Therefore, this study aimed to investigate the potential molecular pathways by which drospirenone and ethinylestradiol, contained in systemic contraceptives, influence dominant *Lactobacillus* species within the vaginal microbiota, and to determine whether these interactions are species-specific or reflect broader ecological dynamics.

## 2 Materials and methods

### 2.1 Strains and cultivation conditions

*Lactobacillus* strains used in this study were *L. crispatus* PRL2021, *L. gasseri* LMG9203 (type strain), *L. iners* LMG14328 (type strain) and *L. jensenii* V94G.

*L. gasseri* LMG9203 (type strain) and *L. iners* LMG14328 (type strain), were obtained from Belgian Coordinated Collections of Microorganisms, and the other strains used were isolated as part of previous work in which written informed consent from each donor was obtained prior to inclusion in the study (Argentini et al., 2022). Each bacterium was initially revived from glycerol stocks and cultivated overnight, anaerobically (2.99% H_2_, 17.01% CO_2_ and 80% N_2_) (Concept 400; Ruskin) at 37 °C, in de Man-Rogosa-Sharpe (MRS) broth (Sharlau Chemie, Barcelona, Spain) supplemented with 0.05% (wt/vol) L-cysteine-HCl.

### 2.2 Impact of drospirenone and ethinylestradiol on *Lactobacillus* spp. growth

To evaluate the hormone susceptibility of *Lactobacillus* strains, cultures were exposed to 12 different amounts of hormones using the broth microdilution method. Starting from initial amounts of 6272 nM drospirenone and 243 nM ethinylestradiol, a two-fold serial dilution was performed to obtain a final load of 3060 pM drospirenone and 126 pM ethinylestradiol. These dilutions were distributed into the wells of a 96-well microtiter plate. The selected range was designed to encompass the reported maximum serum drug concentrations (Cmax)f these hormones in plasma following pharmacological administration (Blode et al., 2012).

Overnight cultures of the strains, grown in De Man–Rogosa–Sharpe (MRS) broth, were diluted to an optical density at 600 nm (OD_600_) of approximately 1.0. A volume of 15 μL of the diluted culture was inoculated into 135 μL of fresh MRS broth containing the appropriate hormone concentration. Experiments were performed in three independent biological replicates for each growth assay. The microtiter plates were incubated under anaerobic conditions at 37 °C for 48 h.

Bacterial growth was monitored by measuring OD_600_ using a plate reader (BioTek, Winooski, VT, USA). Optical density values were recorded at 3-min intervals throughout the 48-h incubation period. Each measurement was preceded by 30 s of shaking at medium speed to ensure homogenization. Growth curves were generated by averaging data from technical replicates.

### 2.3 Exposure of *Lactobacillus* strains to hormones in *in vitro* experiments

All bacterial strains, each representative of CST (Mancabelli et al., 2021), were inoculated into 30 mL of freshly prepared simulated vaginal fluid (SVF) broth (Pan et al., 2020), modified to contain a reduced glycogen concentration of 0.2% instead of the standard 1%, and supplemented with 2 μM (wt/vol) of selected hormones (Zimmermann et al., 2019). Each strain was subjected to four different growth conditions: a hormone-free control and three hormone-treated conditions, corresponding to the presence of either ethinylestradiol (EE), drospirenone (DRSP), or a combination of both compounds (DRSP/EE). For each condition, experiments were conducted in biological triplicate to ensure reproducibility. Specifically, the cells were inoculated to reach a final OD_600_ nm of 0.1. After inoculation, growth was monitored until the exponential phase, and at an OD_600_ nm between 0.6 and 0.8, the cells were harvested by centrifugation at 6,000 rpm for 5 min and stored at −80 °C pending RNA extraction. The same procedure was used to obtain control samples, i.e., the selected *Lactobacillus* strains inoculated in SVF broth without the addition of any hormones. At this point, cells were harvested by centrifugation at 6,000 rpm for 5 min.

### 2.4 RNA extraction and sequencing

Total RNA was extracted following previously established protocols (Turroni et al., 2010). In brief, cell pellets were resuspended in 1 mL of QIAZOL reagent [Qiagen, United Kingdom; (Cat. n: 79306)] and combined with 0.8 g of 10^6^μm glass beads (Sigma). Cell disruption was achieved using a bead-beating procedure consisting of alternating 2-min shaking cycles and 2-min incubations on ice, repeated multiple times. Lysates were centrifuged at 12,000 rpm for 15 min, and RNA was recovered from the aqueous phase. RNA purification was performed using the RNeasy Mini Kit (Qiagen, Germany), in accordance with the manufacturer’s instructions. RNA integrity was assessed via TapeStation 2200 (Agilent Technologies, USA), while RNA load and purity were determined spectrophotometrically (Eppendorf, Germany). For each sample, 100 ng–1 μg of total RNA was treated with QIAseq FastSelect – 5S/16S/23S (Qiagen, Germany) to deplete ribosomal RNA. Following depletion, mRNA yield and quality were re-evaluated using the TapeStation 2200.

RNA libraries were prepared with the TruSeq Standard mRNA Library Prep Kit (Illumina, San Diego, CA) and sequenced on the Illumina NextSeq 500 platform using a High Output v2.5 kit (150 cycles, single-end), generating a total of 118,748,909 reads, with an average of 2,473,936 reads per sample. Raw sequencing data were subjected to quality control, with reads filtered to remove low-quality sequences (mean quality score < 20; read length < 150 bp) and residual ribosomal RNA using METAnnotatorX2 (Milani et al., 2021). This process yielded a total of 104,394,314 high-quality reads, corresponding to an average of 2,174,882 reads per sample. Read alignment to the corresponding reference genomes of the lactobacilli strains was performed using Bowtie2 (Langdon, 2015). Quantification of gene-level expression was conducted with the htseq-count function from the HTSeq package in “union” mode (Anders et al., 2015). Genes exhibiting low expression levels (counts per million, CPM < 1) were excluded from subsequent analyses. Normalization of read counts was carried out using the trimmed mean of M-values (TMM) method. Differential gene expression was assessed using the EdgeR package (Robinson et al., 2009), with results expressed as log_2_ fold change (logFC) between experimental and control conditions.

### 2.5 Bacterial gene expression analysis qRT-pCR

To validate the overexpression of the s*asA_1*, *ribBA* and *ribE* genes in each *L. crispatus* strain, we performed quantitative reverse transcription-PCR (qRT-PCR)-based assays targeting the genes of interest. Reverse transcription of RNA into cDNA was carried out using the iScript Select cDNA synthesis kit (Bio-Rad Laboratories) under the following thermal protocol: 5 min at 25 °C, 30 min at 42 °C, and 5 min at 85 °C. qRT-PCR was performed using SYBR Green technology with PowerUp SYBR Green Master Mix (Thermo Fisher, US) on a Bio-Rad CFX96 detection system, following the manufacturer’s instructions (Bio-Rad). Primers specific to each gene were designed and validated *in silico* using Primer-BLAST (Supplementary Table 5). The qRT-PCR was performed with the following cycling parameters: an initial hold at 50 °C for 2 min, followed by 95 °C for 2 min, and 40 amplification cycles of 95 °C for 15 s and 60 °C for 1 min. Gene expression levels were normalized to the housekeeping genes recA, secA, and 16S rRNA (Rocha et al., 2015). For each reaction, 15 ng of cDNA template was used. Negative controls (no DNA) were included for each primer pair in every run. Standard curves were generated using the Bio-Rad CFX96 software to assess amplification efficiency.

### 2.6 Statistical analysis

Differences in lactobacilli growth in response to hormone exposure were evaluated using nonparametric independent-samples Kruskal–Wallis tests and a One-way Anova (IBM SPSS Statistics for Windows). The edgeR package was used for the comparison of count-based expression data across different bacterial growth conditions (Robinson et al., 2009). Specifically, raw counts were transformed into counts per million (CPM) and log_2_-counts per million (log-CPM) values using the CPM function of edgeR. Genes with low counts (CPM < 1) across all conditions were excluded. The Trimmed Mean of M-values (TMM) method was used to normalize the read counts for variations in library size (sample-specific effect). In the differential gene expression analysis, the log_2_-ratio was used to represent the average log-fold-change (logFC) in gene expression between the reference sample and each test sample. The False Discovery Rate (FDR) procedure was used in multiple hypothesis testing to adjust for multiple comparisons.

## 3 Results and discussion

### 3.1 Effects of drospirenone and ethinylestradiol on *Lactobacillus* spp. growth

**FIGURE 1:**
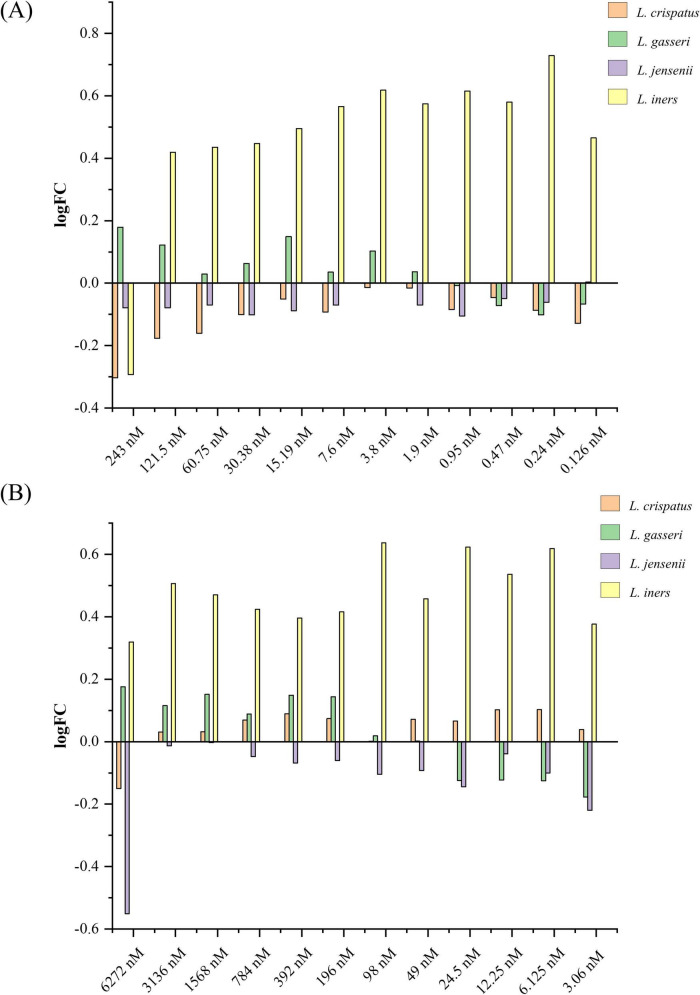
Growth assay of lactobacilli in the presence of different amounts of hormones. **(A)** Represents the logFC of lactobacilli growth in the presence of different amounts of drospirenone. Each pillar shows bacterial growth at the 12 selected drospirenone concentrations, ranging from 6.272 nM to 3.06 nM. **(B)** Represents the logFC of lactobacilli growth in the presence of different amounts of ethinylestradiol compared to the control condition. Each pillar shows the bacterial growth at the 12 selected ethinyl estradiol concentrations, ranging from 243 nM to 0.126 nM. Specifically, logFC values were calculated as the ratio of the mean OD_600_ values measured in the presence of the hormone to those measured in the control condition (growth in the absence of hormone). Data represent the mean of three independent biological replicates for each growth assay.

To investigate whether sex hormones might directly influence the growth capacity of lactobacilli in a vaginal-like environment, we assessed their proliferation in MRS broth supplemented with increasing amounts of drospirenone and ethinylestradiol (3.06–6272 nM for drospirenone and 0.126–243 nM of ethinylestradiol) encompassing plasma levels detected after administration of the contraceptive pill (Blode et al., 2012). Growth variations, expressed as the log_2_ fold change (logFC) relative to the control condition (MRS broth without hormones), ranged between approximately −0.5 and +0.7, indicating moderate effects without evidence of either drastic inhibition or strong stimulation (no value is statistically significant) (Figures 1A,B). Despite this, it is possible to observe that *L. jensenii* showed a slightly negative response to both hormones, *L. iners* showed marked variations as a function of dose, with growth oscillations, and *L. gasseri* displayed a biphasic response, with stimulation at higher amounts and growth reduction at lower doses, regardless of the hormone tested. Finally, *L. crispatus* PRL2021 responded differentially: in the presence of ethinylestradiol, growth reduction was observed at higher concentrations but normalized at physiological plasma levels (around 1.9 nM); in contrast, drospirenone exposure resulted in a more pronounced inhibition at higher concentrations, followed by recovery at lower levels, without displaying a clear dose-dependent trend (Figures 1A,B and Supplementary Table 1).

### 3.2 Effects of hormones on *Lactobacillus* transcriptomes

To understand the direct molecular effects of sex hormones on vaginal lactobacilli, we cultured different *Lactobacillus* species *in vitro* in the presence or absence of drospirenone and ethinylestradiol, synthetic analogues of progesterone and 17β-estradiol commonly used in hormonal contraceptives (Blode et al., 2012), using a medium that mimics vaginal fluid (Pan et al., 2020).

RNA was extracted and subjected to RNA-seq analysis, yielding a total of 104,394,314 quality-filtered reads, with an average of 2,174,882 reads per sample (Supplementary Table 2). Differential gene expression was assessed by considering only genes with a log_2_ fold change of ≥1 and an FDR-adjusted *p*-value of ≤0.05.

**FIGURE 2:**
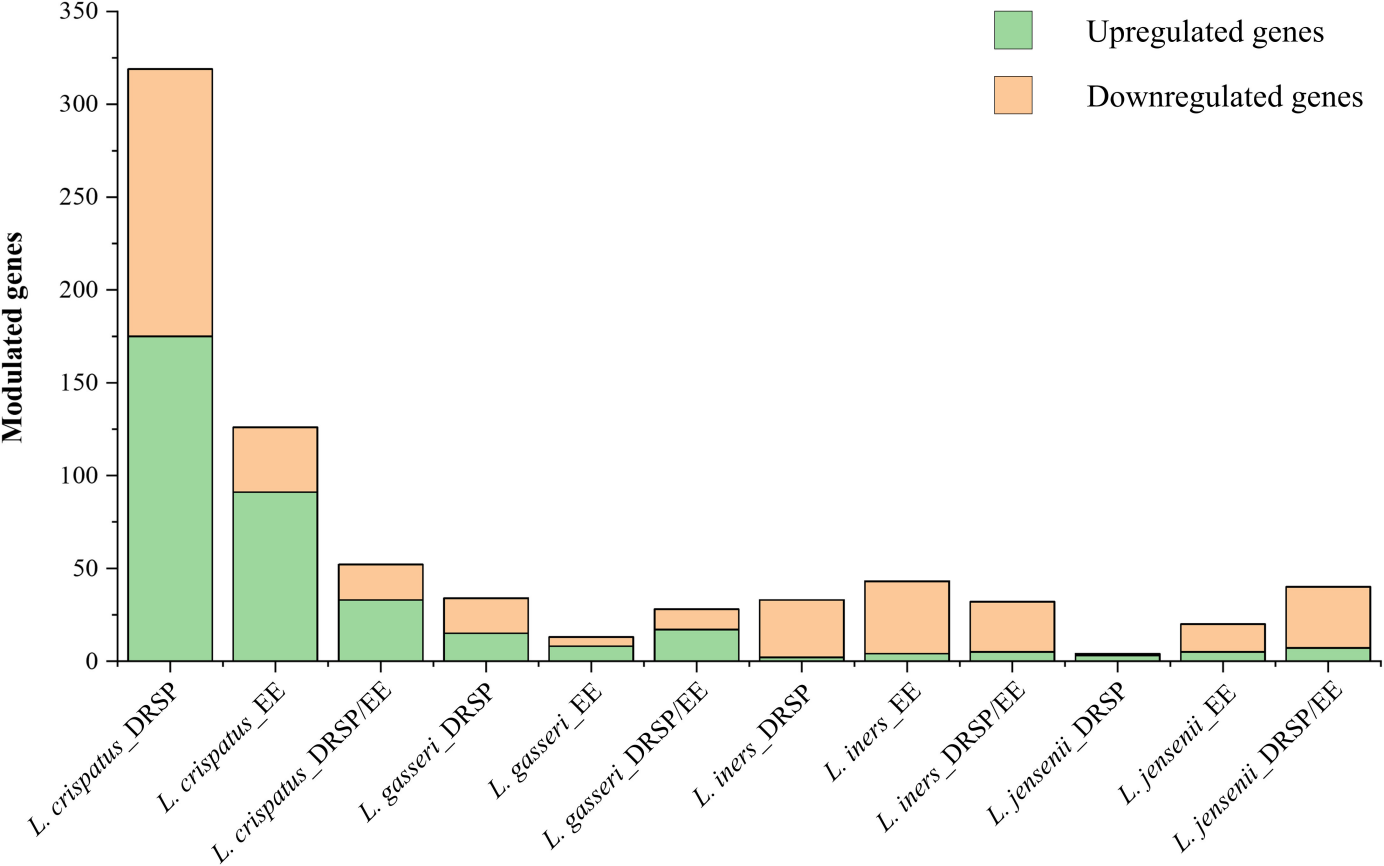
Effects of drospirenone (DRSP) and ethinylestradiol (EE) on the transcriptomes of *L. crispatus* PRL2021, *L. gasseri*, *L. iners* and *L. jensenii*. The figure displays the number of upregulated and downregulated lactobacilli genes under different experimental conditions with the hormones drospirenone (DRSP), ethinilestradiol (EE), and both (DRSP/EE). Data represent the mean of three independent biological replicates for each RNA-seq assay.

**FIGURE 3:**
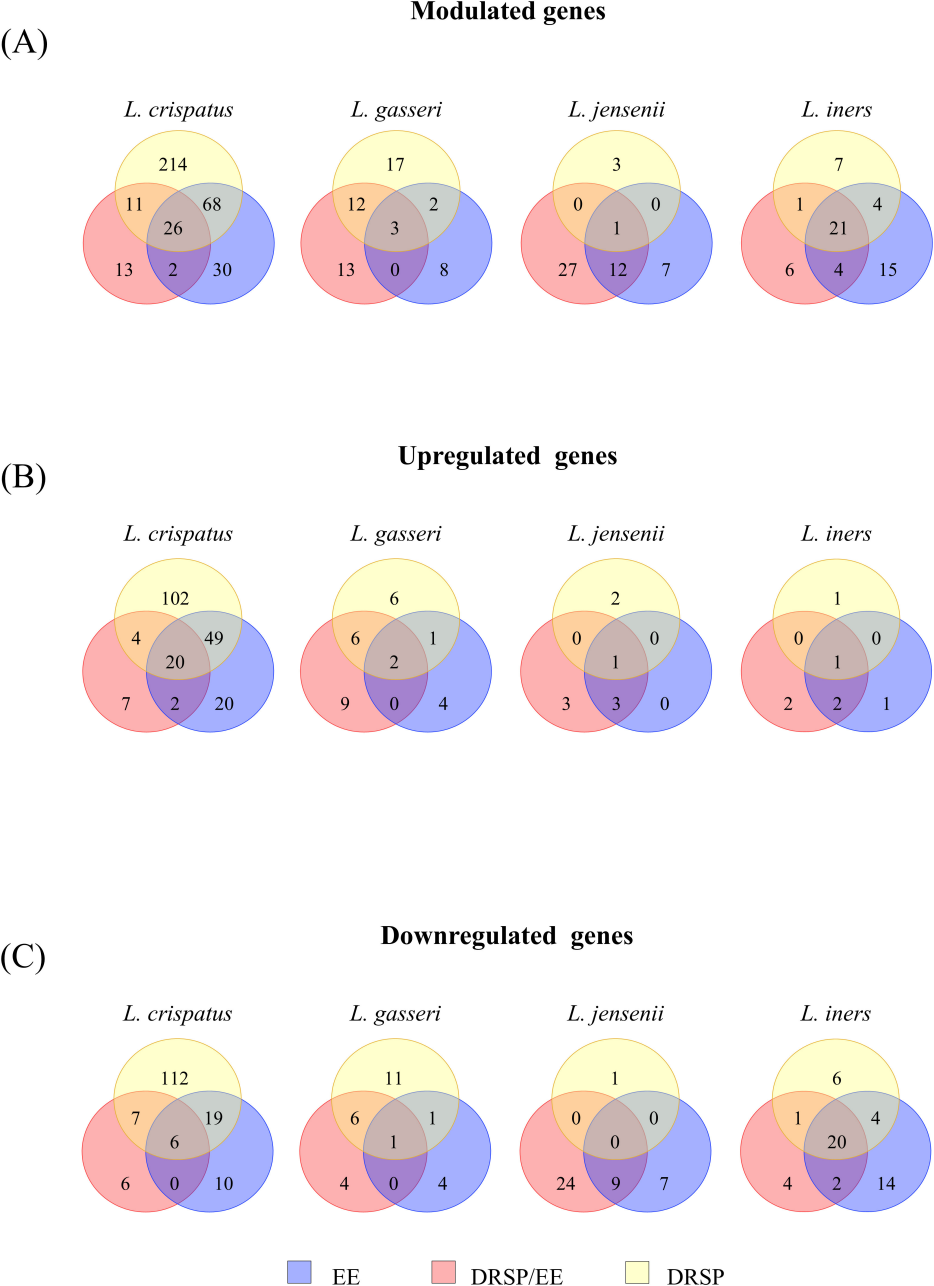
Venn diagrams depicting all modulated genes under the three hormonal conditions. **(A)** Displays Venn diagrams showing the number of shared and unique lactobacilli-modulated genes under different experimental conditions. **(B)** Presents Venn diagrams showing the number of shared and unique lactic acid bacteria upregulated genes under different experimental conditions. **(C)** Presents Venn diagrams illustrating the number of shared and unique lactobacilli downregulated genes in the presence of the hormones drospirenone (DRSP), ethinilestradiol (EE), and both (DRSP/EE).

The number of significantly differentially expressed genes varied across strains and conditions (Figure 2 and Supplementary Table 3). The stronger response was observed in *L. crispatus*, with an average of 100 modulated genes across conditions, compared to 13 in *L. gasseri*, 5 in *L. jensenii* and 4 in *L. iners*. The latter three species displayed fewer differentially expressed genes and a predominance of downregulation, particularly evident in *L. iners* LMG14328, and many of the modulated genes were shared across multiple hormonal exposure conditions (Figures 3A–C).

To better characterize the transcriptional response, we performed a functional categorization of the modulated genes using the Cluster of Orthologous Groups (COGs) classification.

Cluster of Orthologous Group functional classification showed that upregulated genes were enriched in categories related to transcription (COG-K), replication and repair (COG-L), translation (COG-J), carbohydrate metabolism (COG-G), cell wall/membrane biogenesis (COG-M), and a substantial fraction in COG-S (unknown function) (Supplementary Table 4). Downregulated genes were also largely represented in COG-S, with notable enrichment in COG-J and inorganic ion transport (COG-P).

Given the association between *L. crispatus* and the maintenance of vaginal eubiosis (Lepargneur, 2016; DiGiulio et al., 2015) and since the presence of hormones most modulates this taxon, we performed an in-depth functional analysis of the upregulated genes in this species.

**FIGURE 4:**
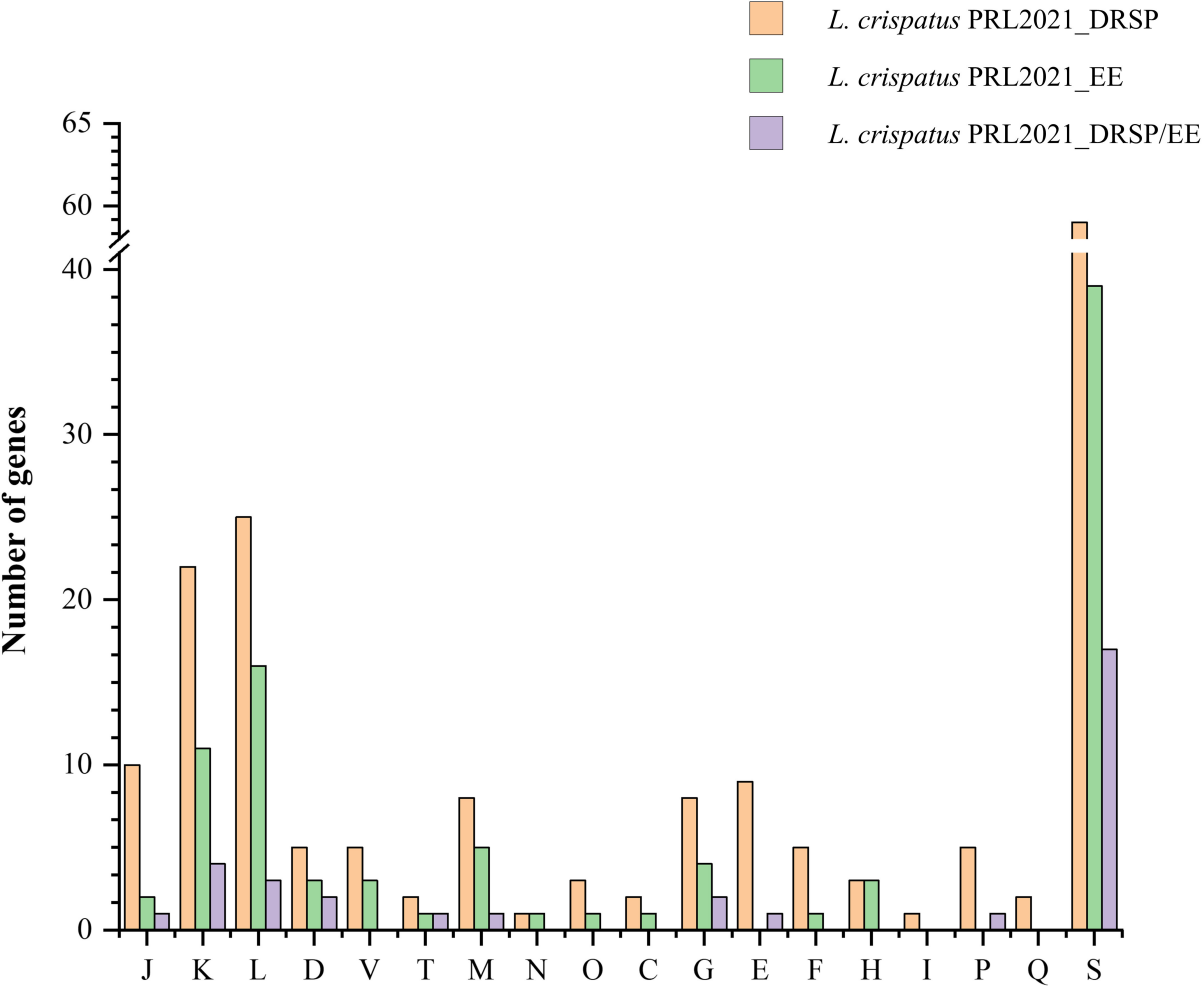
Cluster of Orthologous Group (COG) categorization of the upregulated genes of *L. crispatus* PRL2021 under the presence of different hormonal conditions. The bars represent the number of loci in different functional categories based on functional categorizations established for the clusters of Orthologous Groups (COGs). The COG functional categories are as follows: J, Translation, ribosomal structure and biogenesis; A, RNA processing and modification; K, Transcription; L, Replication, recombination and repair; B, Chromatin structure and dynamics; D, Cell cycle control, cell division, chromosome partitioning; Y, Nuclear structure; V, Defense mechanisms; T, Signal transduction mechanisms; M, Cell wall/membrane/envelope biogenesis; N, Cell motility; Z, Cytoskeleton; W, Extracellular structures; U, Intracellular trafficking, secretion, and vesicular transport; O, Post-translational modification, protein turnover, chaperones; X, Not assigned; C, Energy production and conversion; G, Carbohydrate transport and metabolism; E, Amino acid transport and metabolism; F, Nucleotide transport and metabolism; H, Coenzyme transport and metabolism; I, Lipid transport and metabolism; P, Inorganic ion transport and metabolism; Q, Secondary metabolites biosynthesis, transport and catabolism; R, General function prediction only; S, Function unknown.

In all three experimental conditions, *L. crispatus* PRL2021 showed prominent upregulation of genes involved in transcription (COG-K), DNA replication and repair (COG-L), and unknown function (COG-S). Notably, genes in the COG-S category accounted for 33%, 42%, and 52% of the upregulated transcriptome in the drospirenone, ethinylestradiol, and combined conditions, respectively, likely reflecting the high proportion of hypothetical proteins in this strain’s genome. Upregulation was also observed in COG-M, COG-G, and COG-E categories, suggesting increased membrane biogenesis, carbohydrate, and amino acid metabolism (Figure 4).

Within the COG-M category, both drospirenone and ethinylestradiol exposure induced *murG* expression, a gene involved in peptidoglycan biosynthesis. Notably, under drospirenone treatment, the neighboring gene *murD*, also implicated in peptidoglycan synthesis, was co-upregulated.

In the carbohydrate metabolism category (COG-G), several genes were upregulated across all tested conditions. These included genes involved in the metabolism of arabinose, D-mannitol, glycerate, galactitol, glycogen, phosphoglycerate, and peptidoglycan, such as *araD1*, *mtlF*, *gpmA5*, *garK*, *gtaA2*, *glgX*, *pspB2*, and *murQ*. Additionally, under ethinyl estradiol exposure, we observed an upregulation of a gene encoding a multiple sugar-binding protein (PRL2021_RS04040), which is predicted to facilitate sugar uptake. Genes in the amino acid transport and metabolism category (COG-E) were also upregulated, including *dapX2*, *pepT2*, *thrC*, *argI*, and *dppE5*, suggesting increased amino acid turnover in response to hormonal exposure.

Interestingly, analysis revealed the upregulation of PRL2021_RS10375, which encodes the sensory histidine kinase *sasA*, under all hormonal conditions (Supplementary Table 3). This kinase is part of the *sasA/rpaA* two-component system (TCS) already described in cyanobacteria, which modulates circadian gene expression (Stolyar et al., 2014). TCSs typically consist of a sensor histidine kinase (HK) and a response regulator (RR), acting through a phosphorylation cascade (Monedero et al., 2017). However, very little is known about the mechanisms by which TCSs in lactobacilli function; it has been shown that they are implicated in diverse adaptive activities, suggesting involvement in various physiological processes. In fact, it has been linked to bacteriocin production (Axelsson and Holck, 1995; Diep et al., 1994; Hühne et al., 1996), growth regulation (Morel-Deville et al., 1998), sensitivity to acidic pH, antibiotics (Alcántara et al., 2011), and phosphate transport and metabolism (Hulett, 1996). Concerning adaptive responses, TCSs in lactobacilli have been associated with stress responses (Morel-Deville et al., 1998), resistance to antimicrobial peptides (Alcántara et al., 2011), and quorum-sensing regulation of virulence and competence (Kleerebezem et al., 1997; Nes et al., 1996; Thoendel and Horswill, 2010), as well as adhesion to human cells (Sturme et al., 2005) and biofilm formation (Su and Gänzle, 2014). Consistent with these roles, exposure of *L. crispatus* PRL2021 to drospirenone upregulated the sasA_1 gene, alongside stress-related genes such as PRL2021_RS01520, which encodes free methionine-R-sulfoxide reductase, a key enzyme in the oxidative stress response (Le et al., 2009). We also observed increased expression of genes involved in phosphate uptake (*pstS1*, *pstB3*) and the regulator *agrA* gene (PRL2021_RS10705), which encodes a response regulator of TCS. In *Staphylococcus aureus*, *agrA* is a key component of the accessory gene regulator (agr) quorum-sensing system that modulates, biofilm formation, and other cell density–dependent processes (Wuster and Babu, 2007; Traber et al., 2008). In addition, homologs of this gene have been characterized in some lactobacilli, which are reported to be involved in cyclic peptide production and adhesion regulation, although lactobacilli remain poorly characterized (Sturme et al., 2005).

Under exposure to ethinylestradiol, *L. crispatus* PRL2021 exhibited upregulation of the *ribBA* and *ribE* genes (PRL2021_RS07305; PRL2021_RS07300), which are involved in riboflavin biosynthesis (Supplementary Table 3). Riboflavin is an essential cofactor in energy metabolism and defense mechanisms against oxidative stress (Dricot et al., 2024). It also plays a key role in maintaining vaginal health due to its antioxidant and anti-inflammatory properties, which support the integrity of the vaginal mucosa (Dricot et al., 2024). Riboflavin requirements are known to increase during menstruation, pregnancy, and lactation (Dricot et al., 2024), and the hormone-induced upregulation of its biosynthetic genes in *L. crispatus* may represent an adaptive advantage, enhancing its persistence and beneficial role within the vaginal niche (Dricot et al., 2024). This response may also represent a direct mechanism by which ethinyl estradiol promotes the proliferation of *L. crispatus* within the vaginal environment (Madden et al., 2012; Bastianelli et al., 2021), thereby contributing to the maintenance of vaginal eubiosis (DiGiulio et al., 2015; Lepargneur, 2016). Given their potential relevance to maintaining vaginal health, the upregulation of *sas*A_1, *rib*BA, and *rib*E was further validated by RT-qPCR. Results confirmed their increased transcriptional levels under the tested conditions. In particular, *sasA_1* expression was significantly upregulated during growth in the presence of drospirenone and ethinylestradiol (Supplementary Figure 1).

## 4 Discussion

Drospirenone and ethinylestradiol, respectively, a fourth-generation progestogen and a synthetic estrogen, represent the main components of hormonal contraceptives (Blode et al., 2012) and influence several biological processes in addition to regulating the menstrual cycle, acting on the vaginal microbiota (Burkman et al., 2011; Sitruk-Ware and Nath, 2013).

In this study, we evaluated the potential direct molecular effects of these hormones on vaginal lactobacilli. Recent studies have demonstrated an indirect interaction between ethinyl estradiol and the presence of lactobacilli, due to the promotion of glycogen synthesis in vaginal cells by *L. crispatus* (Madden et al., 2012; Bastianelli et al., 2021). However, the direct mechanisms and implications of these interactions remain largely unknown. In this context, we focused our analyses on four bacterial species, each representative of a typical vaginal microbial community, as the most abundant in the microbiota (Mancabelli et al., 2021).

Growth assay results suggest that drospirenone and ethinylestradiol may differentially modulate lactobacilli growth, likely through threshold-dependent physiological mechanisms or adaptive responses rather than a direct and linear effect on bacterial growth.

Functional genomic analyses show that each strain responds differently to exposure to varying hormone concentrations, indicating that *L. crispatus* PRL2021 is most affected. It is interesting to note that in all three growth conditions the presence of the hormone favors the expression of the gene *sasA_1*, histidine kinase protein, a probable receptor component of TCS in lactobacilli (Stolyar et al., 2014; Monedero et al., 2017), which is associated in the case of the presence of drospirenone, with the upregulation of genes involved in the stress response, phosphate metabolism, and in the synthesis of transcription regulatory proteins. In contrast, in the presence of ethinylestradiol, it is associated with upregulation of genes involved in riboflavin metabolism. This data suggests that since riboflavin has been associated with antioxidant activity and mucosal homeostasis (Dricot et al., 2024), this mechanism may represent a positive feedback loop in which estrogen not only facilitates *L. crispatus* colonization through glycogen availability (Madden et al., 2012; Bastianelli et al., 2021) but also may enhance its functional contribution to the maintenance of vaginal eubiosis via increased riboflavin production. Some genes are up- or downregulated in the presence of hormones but have unknown functions, suggesting the involvement of unknown mechanisms. Nevertheless, these findings provide a solid foundation for further investigations into the functional roles of these genes and, more broadly, into the potential of *L. crispatus* PRL2021 to serve as a key strain in maintaining vaginal microbiota homeostasis and, consequently, supporting women’s health. Overall, our results suggest that sex hormones can modulate gene expression in lactobacilli in a species- and strain-specific manner, likely through direct molecular interactions with bacterial cells. The complex activation of downstream signaling pathways observed in *L. crispatus* PRL2021 may underlie its central role in promoting vaginal health, although this hypothesis warrants further experimental validation to clarify the mechanistic basis of these interactions and their physiological relevance.

Furthermore, using a single strain of lactobacilli as a representative of a synthetic microbial community, while allowing us to control key variables and conduct reproducible experiments, constitutes a significant simplification of the vaginal ecosystem. Furthermore, integrating transcriptomic data with other analyses, such as metabolomics, would have enabled a more comprehensive understanding of the functional consequences of exposure to the hormonal condition.

Nonetheless, our findings provide a valuable starting point for future research into how host-derived molecules, such as sex hormones, may affect the health of the vaginal ecosystem throughout a woman’s life. Our results provide preliminary insights into potential interactions among different domains, including host products such as hormones and microorganisms. These findings suggest that hormones trigger species-specific bacterial responses that may influence ecological suitability and have functional implications for human health.

## Data Availability

The datasets presented in this study can be found in online repositories. The names of the repository/repositories and accession number(s) can be found in the article/Supplementary material.
