# Supplementary figures and images for: Exploring the molecular crosstalk between the sex steroids drospirenone and ethinylestradiol with vaginal lactobacilli
Source: Front Microbiol. 2026 Apr 9;17:1725887. doi: 10.3389/fmicb.2026.1725887 (PMC13102579; doi:10.3389/fmicb.2026.1725887)

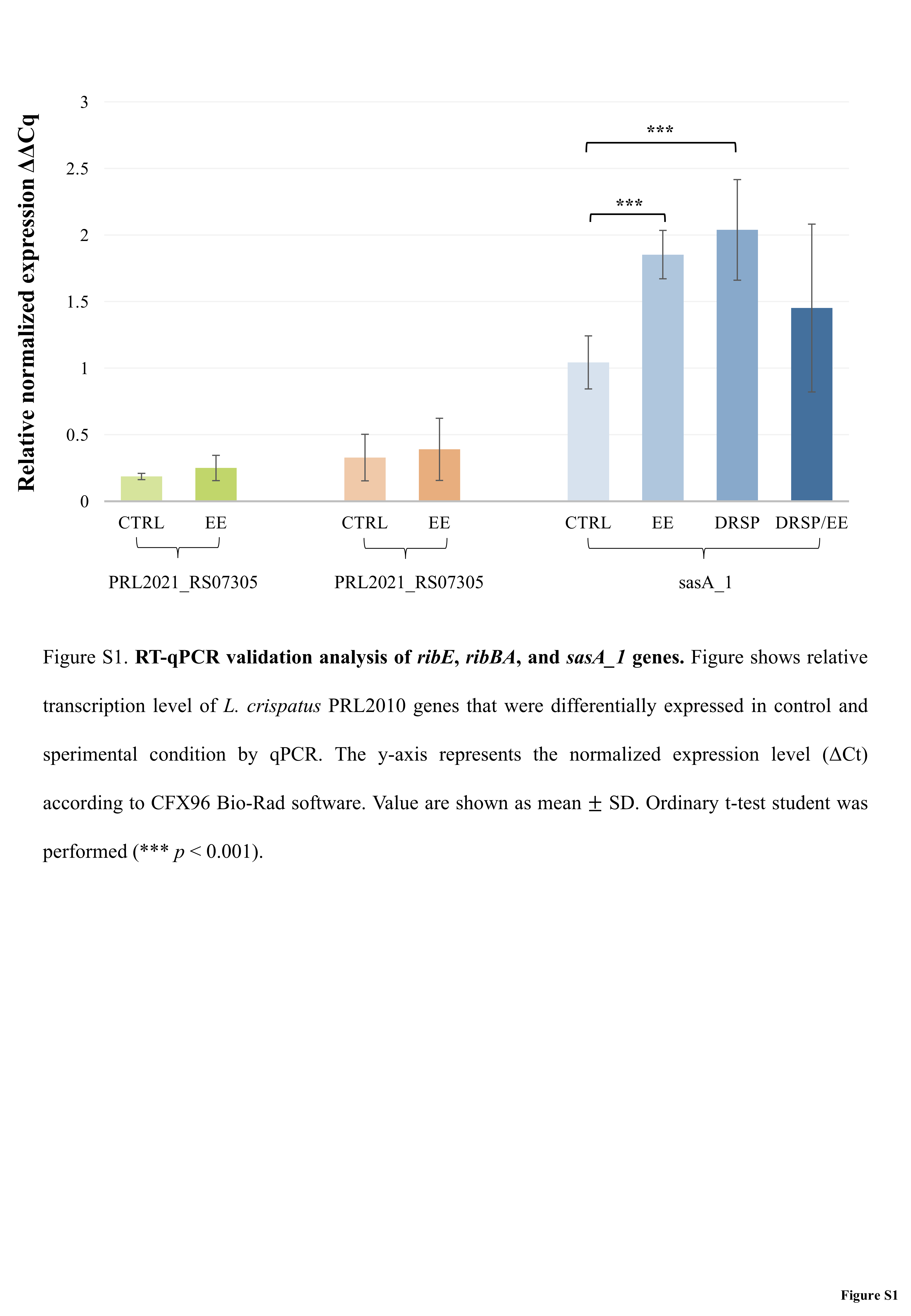

Supplement: Supplementary file 2 [file Image_1.tif]
